# The gendered experience with respect to health-seeking behaviour in an urban slum of Kolkata, India

**DOI:** 10.1186/s12939-018-0738-8

**Published:** 2018-02-14

**Authors:** Moumita Das, Federica Angeli, Anja J. S. M. Krumeich, Onno C. P. van Schayck

**Affiliations:** 1 School for Public Health and Primary Care, Maastricht University, Maastricht, The Netherlands; 2 Centre for Study of Social Change and Development, Institute for Social and Economic Change (ISEC), Bangalore, India; 3 Department of Organization Studies, School of Social and Behavioural Sciences, Tilburg University, Tilburg, The Netherlands; 4 Department of Health Ethics and Society, School for Public Health and Primary Care, Maastricht University, Maastricht, The Netherlands; 5 Department of General Practice, School for Public Health and Primary Care, Maastricht University, Maastricht, The Netherlands

**Keywords:** Formal care, Gender, Health care, Informal care, Health-seeking behaviour, Urban slums

## Abstract

**Background:**

Empirical evidence shows that the relationship between health-seeking behaviour and diverse gender elements, such as gendered social status, social control, ideology, gender process, marital status and procreative status, changes across settings. Given the high relevance of social settings, this paper intends to explore how gender elements interact with health-seeking practices among men and women residing in an Indian urban slum, in consideration of the unique socio-cultural context that characterises India’s slums.

**Methods:**

The study was conducted in Sahid Smriti Colony, a peri-urban slum of Kolkata, India. The referral technique was used for selecting participants, as people in the study area were not very comfortable in discussing their health issues and health-seeking behaviours. The final sample included 66 participants, 34 men and 32 women. Data was collected through individual face-to-face in-depth interviews with a semi-structured questionnaire.

**Results:**

The data analysis shows six categories of reasons underlying women’s preferences for informal healers, which are presented in the form of the following themes: cultural competency of care, easy communication, gender-induced affordability, avoidance of social stigma and labelling, living with the burden of cultural expectations and geographical and cognitive distance of formal health care. In case of men ease of access, quality of treatment and expected outcome of therapies are the three themes that emerged as the reasons behind their preferences for formal care.

**Conclusion:**

Our results suggest that both men and women utilise formal and informal care, but with different motives and expectations, leading to contrasting health-seeking outcomes. These gender-induced contrasts relate to a preference for socio-cultural (women) versus technological (men) therapies and long (women) versus fast (men) treatment, and are linked to their different societal and familial roles. The role of women in following and maintaining socio-cultural norms leads them to focus on care that involves long discussions mixed with socio-cultural traits that help avoid economic and social sanctions, while the role of men as bread earners requires them to look for care that ensures a fast and complete recovery so as to avoid financial pressures.

## Background

Past studies have shown that gender and health are related in many different ways [1–3]. This is also the case when comparing the health-seeking inclination of men and women. In fact, gender relations, gendered notions in terms of health, cultural notions regarding how male bodies function differently than female bodies and gendered differences in access to health care have all been shown to shape differences in the health-seeking behaviour of men and women in many complex ways [4]. Moreover, studies from across the globe show that gender differences with regard to health-seeking behaviour are not only influenced by factors such as power relations [5], structural positions and age hierarchies [6], culturally-prescribed gender roles [7] or economic factors apart from cultural context [8], but also illustrate that the way in which gender and health-seeking are inter-linked is unique for each setting [9], including slum areas [10].

Despite the presence of abundant studies on gender differences in health-seeking behaviour, it would be appropriate to explore these relationships in the context of different cultural settings, given the significant relevance of the socio-cultural context underlying varying health-seeking behaviours. By consequence, the present study investigates how health-seeking behaviour is influenced by gender interactions in slum areas. Urban slums in India differ from other communities because of their complex socio-cultural structure, due to cultural heterogeneity and acculturation [11]. Being migrants, the lifestyle of the people itself changes a lot once they step into the slums, requiring them to make adjustments at every stage and compromise with every situation, resulting into the emergence of an acculturation in perception, attitude and psychological behaviour different from their native place. The impact of religious and cultural plurality compels the people to improvise their values, morals and attitudes. They adopt an attitude that is a mixture of both modernity and traditionalism [12]. Many of the folk practices characteristic of urban slums is functional in coping with the disoriented and disorganised social conditions of industrialisation and urbanisation. Previous studies have shown that the health of slum populations is much worse than in other urban areas [13]. Also, the public health impacts of health problems in slum areas are immense. As health and illness are identified, defined and categorised by culture based on people’s socio-cultural contexts and prior experiences, therefore, in order to discern the true nature of health and illness of the slum dwellers there is a need to understand from their perspective the mechanisms through which culture govern the decisions about recognising and evaluations of treatment. The contextual nature of gendered health-seeking practices like approaching other healthcare resources available in a given area also stems out simultaneously [14]. Understanding gender differences in respect of therapeutic choices in the slum context is crucial to developing appropriate policies to promote and provide suitable treatment sources for women’s and men’s requirements and thereby ensure a better utilisation of health care facilities.

## Literature review

Several publications in the area of gender and health have established that gender differences exist with respect to decision-making regarding the appropriate type of treatment. Some studies have found, for example, that women in developing countries utilise formal health care to a lesser extent than men [15] and instead are more inclined towards traditional healing options [16]. According to Harrison and colleague [17], the gender socialisation process, which is in itself shaped by the socio-cultural ethos, also tends to impact health-related notions and habits, including decisions regarding when and where to seek help.

Gender also leads to differences when it comes to symptoms of illness expressed, social support mobilised thereafter and the socio-cultural ethos that tends to impact access to appropriate treatment and care. Studies examining gender differences in the experience and expression of symptoms propose that women not only ask for increased social support compared to men, but also report significantly higher rates of distress [18, 19]. This may be partly explained by the fact that in certain societies social interactions and the exhibition of communal behaviour are considered as part of the feminine role, while the masculine role involves independency and giving prime focus towards familial responsibilities rather than looking into community needs [20]. In contrast, Macintyre et al. [21] consider that men are more willing to report and seek help for common cold complaints than women. They claim that such health-seeking behaviour emerges because illness among men is not easily acknowledged by professionals and that a clinical score based on a highly objective set of criteria, like quantity of nasal secretion, nature of swollen glands and so forth, is required to make their illness eligible for treatment.

Gender relations not only influence decisions regarding the expression of symptoms or distress and treatment, but also tend to create other societal obstacles in accessing health care for women. Vlassof [22] shows, for instance, that women’s inferior status in family and society restricts their access to health care, decision-making, education and economic resources. As a result, women remain uninformed about health issues, fail to acknowledge illness or depend on older family members or men for receiving health care. This is more visible in countries where such structural constraints do not permit women to pay attention to or to seek health care services for their illnesses [23].

Finally, gender differences may influence actual access to health care, including treatment. Although accepting sickness and getting treatment are more socially acceptable among women, they cannot easily avail themselves of treatment for an illness that is equated with social consequences. A study [24] involving Caucasian men and women shows that women seeking treatment for alcohol addiction face greater social resistance than men. Besides, they are more likely to meet with opposition and a social penalty from family and friends for seeking general health care compared to men, who rarely face such opposition.

The above literature review suggests that gender and health-seeking behaviour can be linked in both direct and indirect ways. The way in which gender shapes health-seeking and access to health can, depending on the social and cultural context, work out positively as well as negatively for either gender. Studies on gender differences in health-seeking suggest that the situation in India is no different. In the Indian context, gender interactions and their inter-linkages affecting therapeutic behaviours in different rural settings have been explored intensively. For instance, studies examining health-seeking patterns across gender in the rural Indian states of Uttar Pradesh, Pune and West Bengal find that due to traditional gender preferences, cheaper public care providers are sought by households for girls, while for boys private qualified providers are consulted, more money is spent and greater distances are travelled, if necessary [25–27].

A few quantitative studies report gender differences in health-seeking behaviour in Indian urban slums [26, 28]. However, qualitative accounts of the complex gender relations affecting therapeutic choices behaviour are still missing. An in-depth understanding of the reasons underpinning gender differences is required, considering the different ways in which gender and health-seeking have been shown to be directly and indirectly related, and considering the contextual and cultural nature of these relationships. This qualitative study in line with emic perspective will enable to understand in-depth the combined influence of socio-cultural context, respondents’ perceptions and self-constructed meanings [29–32] behind their behavioural patterns in healthcare utilization. Moreover, the unique socio-cultural context of India’s slums characterised by heterogeneous and dynamic populations [33], can be expected to considerably differ from other (Indian) settings.

While placing the social context at the forefront of the gender and health-care agenda, this study aims to provide fresh insights into gender differences in health-seeking behaviour in the Sahid Smriti Colony, an urban slum in Kolkata, India. The key questions addressed in this study are: (1) Do gender preferences exist in making choices among the different available therapies in India’s urban slum settings? (2) If yes, what kinds of therapies are used by men and women in slum areas? (3) How do complex gender interactions function in slum settings and influence the therapeutic behaviour of men and women?

## Methods

### Study site

An exploratory study was conducted on slum dwellers in Kolkata during August–September 2012. According to the Kolkata Municipal Corporation (KMC), out of a total of 141 municipal wards, slums are spread in 138 wards. Out of these slums, Sahid Smriti Colony, situated at Baghajatin, under Kolkata Municipal Corporation (KMC) jurisdiction was selected. There were two reasons for selecting the Sahid Smriti Colony slum. Firstly, it is a peri-urban slum located on the south-eastern outskirts of the city. Being in a process of acculturation, a glimpse of both traditional and modern ways of life can be witnessed in a peri-urban slum [34]. Secondly, such a transition is also witnessed in health practices. In Sahid Smriti slum, both folk and alternative healing practices exist and are utilised alongside formal health care. This slum was selected to see how complex gender interactions function within this setting in establishing preferences among these two domains of therapy.

### Participant recruitment

The participants consisted of 34 men and 32 women living in the study slum. The characteristics of the 66 study participants are shown in Table 1. Given the linguistic, religious and cultural heterogeneity, an extensive sample was selected based on the following criteria: (1) belong to any of the three major religious groups predominant in the study slum, namely Hindu, Muslim and Christian; (2) over 16 years of age and limited to adults; (3) are native speakers of the Bengali language; (4) can clearly recall their experiences with regard to the causes, nature of treatment and provider sought; (5) consent to re- interviews, if and when necessary, during the study period; and (6) have been resident within the study slum for at least five years (people residing less than five years were mostly refugees). The sample size was decided following Daniel Bertaux’s [35] concept of ‘theoretical saturation', which states that data saturation in a qualitative study typically occurs by the time 12 interviews have been analysed. Additional new patterns emerge rarely after that, as 12 individual interviews are enough to include 88–92% of information [36]. Based on this recommendation in relation to the saturation threshold, the present study was based on an initial selection of 12 men and 12 women each from three religious groups in the study area, with a total of 72 participants. However, men and women could not be selected on an equal basis because of absenteeism or unwillingness to participate or refer to anyone. A total of 66 participants, i.e. 34 men and 32 women, agreed to participate. Mostly, after the eighth interview (of each man and each woman in the three religious groups) no new shared themes were generated from the interviews. Therefore, it was deemed that the data collection had reached a saturation point based on the data saturation model [37, 38]. Four more interviews for data collection with Hindu and Muslim religious groups and two more interviews with Christian male participants^1^ were carried out to ensure and confirm that no new themes emerged, but only instances of the same themes [39–41]. The snowballing technique was used to select participants, as everyone in the study area was not comfortable in discussing health-seeking behaviour freely [42]. Initially, an ex-municipal medical officer (personally known to the first author) was approached because of his good contacts with some health workers residing in the study slum. Sampling was therefore facilitated through one of these female health workers, using her knowledge about the slum dwellers. She introduced the first researcher to two women and three men. These initial five participants were asked to recruit participants of their own gender who not only met the inclusion criteria, but also were willing to discuss freely issues associated with treatment choices.

**Table 1:** Socio-demographic profile of participants in the study slum

Particulars	Sahid Smriti Colony slum (*n* = 66)	
	Male (*n* = 34)	Female (*n* = 32)
Age groups		
16–30	10	17
31–45	19	11
46 and above	5	4
Marital status		
Married	25	26
Unmarried	8	3
Widow	0	2
Widower	1	0
Separated	0	1
Educational status		
Illiterate	9	12
Literate	25	20
Origin of the population		
Rural Kolkata	24	27
Within Kolkata	5	2
Bangladesh	5	3
Social groups		
General	10	7
Scheduled Castes (SCs)	21	19
Scheduled Tribes (STs)	3	6
Religion		
Hindu	12	12
Muslim	12	12
Christian	10	8
Employment status		
Full employed	16	1
Contractual	11	0
Unemployed	7	31

Source: Based on data collected in the earlier phase of the field study

### Data collection

In-depth face-to-face interviews were conducted with the participants by using a semi-structured interview guide with probing questions to keep the participants on track and also allow them to structure the interview by themselves, so as to bring forth the issues that were important to them. The questions were open-ended, so that the interviewer could probe more on particular concepts of interest to the study. The interviews focused on exploring different health choices influenced by gender in terms of the range of therapies utilised, factors influencing health-seeking decisions and the process of evaluating the efficacy of treatment from a gender viewpoint. Each face-to-face interview was conducted in a private room either in the participant’s house or in the workplace. At the moment of data saturation (that is, when no new data emerged and the existing literature did not add any new information), the data collection was ended. Interviews were later transcribed and translated into English. The interview guide was first created and piloted in English. After revisions and further piloting, the research team then translated the interview into Bengali. The translation was contextual rather than literal, meaning that questions were translated to relay the best meaning in colloquial spoken Bengali. Following a round of piloting with the Bengali interview guide, the questions were then back-translated into English to maintain consistency of meaning between the two versions. After conducting five pilot interviews in Bengali, the questions were further refined and finalised to best convey the essence of the questions. Each interview lasted around 45 to 60 min. All interviews were audio recorded, transcribed and translated into English. Since the field data was collected by the main researcher, a woman, this favoured openness of female respondents, who are traditionally more reserved. However, it may have introduced a bias in gathering the male perspectives and therefore adds a limitation to the study.

### Data analysis

The in-depth interviews that were first audiotaped were later on written down word for word. Prior to that, the recordings were listened to several times to ensure the accuracy of the transcription. The transcribed data was then read and re-read several times. The transcripts were translated from Bengali into English. Professional help was sought out to examine the translations of the original text. Some corrections were made and inconsistency was avoided. Our analysis used descriptive codes to generate thematic concepts [43]. The interviews with female participants were coded separately from the interviews with male participants. This approach was chosen to inductively grasp the difference between male and female experiences, in line with the aim of the study to investigate the ‘gendered experience’ of health-seeking behaviour.

The first author conducted the initial thematic analysis under the supervision of a sociologist (local facilitator) with extensive qualitative methods expertise. Briefly, the thematic analysis includes several phases: familiarise yourself with your data (team), generate initial codes (first author), search for themes (team), review themes (team), define and name themes (first and second author) and produce the report (team). The initial codes were framed by constantly moving back and forward between the entire dataset. These codes identified features of the data that the first author considered pertinent to the research question. After identifying a list of codes, these were sorted into potential sub-themes by combining different codes. Sub-themes were constantly compared and refined further, bearing in mind the aim of the study. The final themes were defined by focusing the analysis on a broader level and refined again. The real meaning of what each theme dealt with was captured and a satisfactory final thematic chart was developed by making clear and identifiable distinctions between the themes. The final phase consisted of developing a set of fully worked-out themes and writing the report. The reliability of the final themes was confirmed by choosing examples from the transcript to verify whether they illustrated elements of the themes. Issues within the themes could be clearly identified when compared with these extracts and thereby presented a coherent account of the point being made.

### Ethical considerations

Because of the non-clinical and non-invasive nature of the study, this research has thoroughly followed the ethical guidelines framed by the National Committee For Ethics In Social Science Research In Health (NCESSRH)^2^^,^^3^ [44–46]. In line with these guidelines, oral consent to participate was obtained from all the participants, after the purpose of the study had been explained, and anonymity and confidentiality were assured. It was specifically explained that comments would not be attributed to a named individual without permission. Written consent was not sought in order to favour an atmosphere of trust, intimacy, and informality, which was believed to create the necessary conditions for the respondents to feel at ease and respond openly and truthfully. The Institute for Social and Economic Change (ISEC) in Bangalore, India, endorsed the project and functioned as a partner institution on site. The ISEC’s Ethics Committee formally examined the study protocol and provided ethical approval.

## Results

We categorised health care facilities into two broad groups: informal and formal care. The former refers to medically unlicensed practitioners [47] and the latter to medically registered [48] and government-licensed alternative medicine practitioners. Table 2 presents a summary of the different types of health care practitioners under informal and formal care. Table 3 shows the pattern of informal and formal health care usage by male and female participants.

**Table 2:** Types of health care practitioners in Sahid Smriti Colony

Informal care	Description	Formal care	Description
Herbalists	Unlicensed traditional medicine men skilled in dispensing herbal medicines	Medical doctors	Qualified allopath with a medical license
Fortune tellers	Illegal practitioners involved in the practice of predicting and giving spiritual explanations about a person’s life	Para-professionals	Medical assistants with a three-year medical training
Shamans	Illegal practitioners involved in healing through magico-religious means	Homoeopaths	Recognised as one of its national systems of medicine by the Indian government
Ritual experts	Priests of the local temple who perform chanting for the well-being of individuals and are not legitimised in the health care system		
Unlicensed drug dealers & drug stores	Drug sellers who do not hold the registration certificate of a pharmacist (d.pharm) and usually dispense medicines without a doctor’s prescription		

Source: Based on data collected in the earlier phase of the field study

**Table 3:** Pattern of formal and informal care usage by men and women

Therapies	Men *N* = 34 (%)	Women *N* = 32 (%)
Formal	12 (35)	0
Informal	7 (21)	15 (47)
Partial use of institutions (formal + informal)	0	10 (31)
Have not used	15 (44)	7 (22)

Source: Author’s calculation based on data collected in the earlier phase of the field study

### Women’s perceptions regarding informal and formal health services

#### Cultural competency of care

The way in which different health care practitioners offer their services determines women’s health-seeking behaviour. Formal practitioners focus only on treating the biological symptoms of illnesses without considering the associated socio-cultural issues. Most of the female participants refer to marital insecurity, financial hardship and exclusion from social festivals, family and the community as (potential) consequences of seeking professional health care without any justifiable causes. Scared as they are of social sanctions, women initially seek informal types of care that are more socio-culturally acceptable in case of any illness. Informal healers are generally competent in dealing with the financial and socio-cultural hassles (besides illnesses) that women experience when they seek treatment, thus securing their position both in the family and society. As a female participant said:

‘No matter what happens [illness], I consult him first. When I do this, two things happen: firstly, I receive treatment, and secondly and most importantly, I can avoid various obstructions [socio-cultural] that we women generally face.’ [user of herbalist, ritual experts and shamans]

Female participants report that only if care in the informal domain fails to show any positive results and creates a life-threatening situation (which is considered a substantive cause) are they allowed to seek formal care, irrespective of its cultural insensitivity. As a woman said:

‘Western medicines act well upon us even when the illness is in its last stage.’ [user of para-professionals, homoeopathy and herbalist]

Lastly, it is observed that social-cultural norms also have a strong impact on women in terms of influencing their health beliefs and habits. This is quite evident in that women do not entirely stop consulting informal healers, even while they seek formal health care. As a woman stated:

‘I will not leave them (informal healers) even when I am using Western medicine. My mother taught me to always respect them.’ [user of herbalist, fortune tellers, ritual experts, homoeopathy]

#### Easy communication

Female participants always tend to look for a treatment that makes them feel comfortable and satisfied during therapeutic interactions. Informal healers sometimes happen to be distant kin of female participants and their families with the same socio-economic status, so they are closely associated. A woman said:

‘They belong to ekisampradaya [same class and caste]… we can readily relate to them.’ [user of herbalist, ritual experts and shamans]

As a result, women find communication a mutually comfortable process, in which they feel at ease while sharing their health-related experiences. As a woman said:

‘They take long hours to discuss in detail and clear our doubts about the cause and nature of the illness.’ [user of herbalist, ritual experts, fortune teller and shamans]

Besides healers, informal practitioners are generally good counsellors. Being usually old and wise, they help women escape pressures caused by family or marital disputes. According to a woman:

‘Whenever problems arise in my family, I first think of him for getting consoled. I know he is there to resolve the tension.’ [user of ritual experts and shamans]

By contrast, women usually avoid approaching formal practitioners because of the absence of smooth and clear interactions caused by the professional and unfriendly behaviour of doctors. As one woman said:

‘Their facial expressions are so cold and confusing; we wonder whether they have really understood our problems.’ [strong believer and user of herbalist, ritual experts, shamans and occasional visitor of medical doctors]

Several women feel unhappy when the nature of their illness is not explained to them in a simple way. Doctors instead use medical terms to explain things, which makes it quite difficult for them to understand. One woman commented:

‘I feel very annoyed because although he [doctor] can easily and in a simple way explain things in our language, he unnecessarily uses all those English words and deliberately confuses us to see the fun.’ [user of herbalist and ritual experts and visitor of para-professional for illnesses that fail to get cured by informal healing]

Naturally, women feel discouraged to seek medical help from formal practitioners, considering it as an unfamiliar therapy.

Some women have a strong feeling that doctors express ‘disgust’ when the patients’ family speaks for her. One woman describes how the clinical doctors react when her husband speaks on her behalf during interactions:

‘They will give an annoying look if my husband wants to explain things as if it is not his business to interfere.’ [user of herbalist, ritual experts and fortune tellers, usually avoids medical doctors unless illness becomes extremely serious]

Moreover, women generally feel that during the patient-doctor interactions family members are more proficient in speaking on their behalf. This is because they think their preconceived notion about clinical care as strange therapy prevents them from discussing health problems freely.

#### Gender-induced affordability

The lack of independent financial means often indicates the starting point for women when considering what type of treatment to seek. Most of our female participants did not earn their own incomes and as such, lacked the financial autonomy and decision-making power when it comes to seeking formal care. Men are the main providers of money for women and, therefore, make all the health care-related decisions for them. However, it is to be noted that men’s decisions are often not in line with women’s preferences. Women discussed several difficulties they faced with men because of dependence on them while trying to seek formal health care. An important obstacle they mentioned is that husbands often suspect that they (women) fake illness. Furthermore, many women remarked that men often do not consider women’s health problems as serious. As a woman stated:

‘He said this is very normal and I am overstressing. Believe me, it was hurting for more than a month, but he was not ready to understand.’ [user of herbalist, ritual experts and homoeopathy]

To avoid such hassles, women find it easier to access informal practitioners as alternatives who generally do not demand money for their services and are willing to accept payment in kind. As observed by a woman:

‘I had no money to pay for the treatment during that time. I was left with some rice and gave it to him. He accepted it gladly.’ [user of herbalist and shamans]

Most of the women expressed that they do not make use of concessions regarding health expenses due to lack of knowledge and information about accredited health care beneficits. As a result, they end up spending huge amounts of money for tertiary care. As they pay out of pocket, they face a terrible financial crisis which they are then unable to recover from for several years. Such non-utilisation of health services, despite their accessibility, happens because of information asymmetry. As one woman expressed:

‘It will be really helpful if they come in person to every door and explain… instead of passing information to only a few in a community meeting.’ [user of herbalist, ritual experts and homoeopathy]

Some women expressed that if each of them had been fully informed of health beneficiaries, they could have avoided financial problems. This, in turn, would have encouraged everyone to use formal health care extensively. By contrast, women said that they had enough information about various informal healers, their kind of treatment charges and concessions. This is due to a wide networking system that informal healers have both at the individual and social levels, which clinical providers lack.

When informal care treatment fails, some women feel the urgency to approach formal care providers. However, they still look for options within the formal care domain that limit monetary loss. As a result, women are seen using homoeopathic care rather than allopathic medicine as an initial choice (even though it is not always effective), primarily because of cheap consultation fees and medical expenses.

#### Avoidance of social stigma and labelling

The fear of being labelled seems to play a major role in determining the choice of therapies, even if it means compromising on the quality of care. As one woman stated, there are some specific illnesses such as contagious skin disease, barrenness and female sexually-related problems that create social stigma apart from severe physical, social and emotional problems for both the patient and the family. It is, therefore, important for every women respondent to see that their illness does not bring any disgrace either to them or to their families. Although women do not deny their illnesses and look for treatment, they do seek therapies that are generally of inferior quality (less effective and slow or with a slight improvement) but that primarily help them escape social stigma and illness-related consequences. In this context, women find informal healers more responsive to their health needs than formal care providers, as some of them assert:

‘It’s far better to seek some other options for care within the community, even if it does not work, than listening to this nonsense and bearing it every time we step out of a clinic.’ [strong admirer and user of herbalist, ritual experts and fortune tellers]

Familiar with this kind of social stigma, informal healers prefer to see only one client at a time. Different timings are allotted to each of the patients in order to protect their identity, thus helping the care seekers avoid becoming the victims of harassment within their community. One woman shared her experience about the abortion of a defective foetus identified after scanning:

‘He [husband] asked me to contact any folk healer from my native place, as he does not want anyone here to know about the problem.’ [preference given to herbalist, ritual experts and shamans, often uses homoeopathy]

By contrast, many women respondents complained that doctors do not care to value the feelings of the patient and family, that there is no privacy during consultations and that doctors see too many patients at a time, thus increasing their chances of meeting a known face and the subsequent risk of secrets getting revealed. A female participant gave an instance of a physician breaching privacy by talking openly in front of others about her illness:

‘I felt so embarrassed that during that moment I thought of running away from the place… He started asking loud enough for others to hear: “Are you taking your leprosy medicine regularly… Are you keeping the infected area clean…”.’ [strong admirer and user of herbalist, ritual experts and fortune tellers, denounces and avoids medical doctors and para-professionals]

#### Living with the burden of cultural expectations

Women expressed that they can easily accept any care, provided it does not breach the cultural norms prescribed for women and disturb their normal activities in life. Cultural norms greatly influence women’s choice of therapies. Women are taught from early on in life to avoid bodily contact with unfamiliar men or known men outside relations, men with whom they share incest and affiliate relations.^4^ Direct physical contacts with these men are culturally proclaimed as a sexual association and women’s position in society is marked as impure and unholy. In order to protect their own pride and good name in the society, therefore, many women deliberately downplay the importance of formal heath care, saying that its professionals violate honour codes relating to sanctity. They comment on the uncaring diagnosis process of the formal care system, that informal healers are much more sensitive while diagnosing the nature of the illness, thus handling women clients with care. As evidenced by a woman’s version:

‘I consulted a doctor (for sexual related problems)… He wanted to examine my private parts. I know they are right and do an accurate analysis. But that was very embarrassing… It was my first and last visit. Baba just touched my wrist, that’s all and explained everything right, from the cause to the required treatment. At the end of the meeting with Baba, I felt clean and that really meant a lot to me.’ [prefers and seeks care of herbalist and ritual experts]

#### Geographical and cognitive distance of formal health care

For women, apart from financial and socio-cultural factors, seeking treatment is also determined by suitable access to care. Women pointed to geographic inaccessibility as one of the factors giving rise to problems such as time constraints, increased travel costs and failure to attend follow-ups. Women often find it difficult to adjust their household activities schedule to accommodate inconvenient appointments (mainly during the daytime) at hospitals and private clinics. As a consequence, they are forced to skip medical check-ups. Furthermore, travel costs discourage women from seeking formal treatment. As remarked by a participant:

‘Within the city, it takes hours to travel from one place to another and simultaneously you have to bear travel costs. It’s like wasting the whole day and money.’ [preference is given to herbalist, ritual experts and shamans, often uses homoeopathy, but avoids clinical care that usually incurs travel and consumes time]

Restricted mobility is not only related to financial issues, but also linked with cultural dimensions that limit access to formal treatment. Hospitals in the study area are situated at a fair distance and women are not allowed to frequently commute alone without company. Family members are not always available to accompany them to hospitals, so they find it difficult to continue their treatment. As a woman said:

‘My sister-in-law and I used to accompany each other [in visiting clinics] before her marriage. But now I often skip my follow-up treatments… My husband has no time to accompany me to the hospital.’ [preference given to herbalist and shamans, is discouraged to use clinical care that usually incurs travel and consumes time]

Thus, in spite of being aware of the fact that formal care is effective, they undermine its value, thinking that at some point in time they will have to face mobility restrictions, resulting into abandoning the treatment before its conclusion. As expressed by a woman:

‘Though Western medicines are good, it is useless if the treatment is withheld from time to time while you are not totally cured.’ [user of herbalist and shamans, is discouraged to use clinical care that usually incurs travel and consumes time]

The non-availability of proper health care infrastructures also reduces women’s interest in formal treatment. Patients are often referred to faraway hospitals due to a lack of sufficient staff, medicines, beds and medical equipment. One woman expressed her inconvenience because of such referrals:

‘We have already spent lots of money during the treatment… Suddenly they said they didn’t have a particular machine and referred me to a different hospital. Again it started afresh; wasting more money and time which we really can’t afford.’ [preference given to herbalist, ritual experts and shamans, is discouraged to use clinical care that usually incurs financial constraints and consumes time]

Because of all these problems which women face while seeking formal care, they tend to look for informal care. This has several advantages: healers are found within reach; no travel costs need to be borne; and healers can be accessed any time when convenient, which, in turn, minimises the chances of discontinuing the treatment.

### Men’s perceptions regarding informal and formal health services

Men believe that they differ from women both in terms of biological and environmental features, so for themselves, they find formal care more suitable than informal health care. The majority of them used the term ‘hard diseases’ to characterise their illness. By this, they mean chronic and communicable diseases. According to men, women nag too much about illnesses, so they are given all sorts of light diseases (i.e. mundane and infectious ailments) as hard diseases cannot be handled by them. For instance, one man commented: ‘Since we are strong, we are given the hard ones to fight with.’ Another man remarked:

‘These women generally have the illness that comes and goes and therefore they are not that dangerous. Quite justified! They nag too much and they get these easy-going ailments which can be easily tackled. And they come to those healers who can easily impress these women by their magic. These illnesses are nothing but those which actually don’t require any medicine. Ours is the real and hard one, these are not that easy to locate and therefore these healers fail to fool us. We require real treatment.’ (strong believer and user of formal health care)

Almost every male respondent normally tended to underuse health care services or believed in delayed help-seeking. This is because they believe they can handle ‘light diseases’ or general health problems. The majority of them responded that they do not seek help for general health problems, however; they look for help for specific and longstanding illnesses. Some of the comments put forward in this background as stated by the men: ‘treatment was not necessary’ since ‘minor illnesses can be fought off’, ‘general illness is not illness, they come and go’. The views of men have been put forward as themes presented below.

#### Ease of access

Most of the men prefer purchasing drugs from drug stores for general ailments, since drug stores are easily accessible within their neighbourhood. This avoids the need for travel, which they usually consider a waste of both time and money. One man, for instance, commented:

‘I usually allot Rs.200 for my medical expenses. If I have to travel to hospitals located far away to meet good doctors, then, out of the allotted money, I have to spend a minimum of Rs.70 on travelling alone. The money left is not enough to get good treatment.’ [self-medication by using medicines from drug stores]

Besides travel costs, the availability of services at any time of the day also influences men’s preference towards unlicensed drug dealers. Drug dealers usually reside within the community, close their shops late at night and can be approached at any time. One man commented:

‘One day my son was suffering from fever in the middle of the night… When we realised that his temperature got very high, immediately I went to Jayanta’s [drug dealer’s] house and got medicine for my son.’ [user of medicines from unlicensed drug dealers and drug stores because of accessibility]

Most of the men do not reconsider their inclination towards drug dealers when it comes to comparing their quality of treatment with that of health professionals. Some men consider the medical knowledge of pharmacists as equivalent to that of clinical professionals. Hence, men feel convinced that they can access quality care from drug dealers as an alternative option. According to a man:

‘I just have to tell them [drug dealers] my problems and that’s all. They give all the directions like a doctor as for how and when to use [medicines]. It gives a feeling of being treated by medical doctors.’ [strongly believes in the efficiency of drug dealers and takes their medicines]

Many others approach drug stores for common illnesses, as no consultation fees are required due to the informal nature of their service. A few others expressed that pharmacists help them save money on medicines by suggesting cheaper medicines (with similar effects) instead of buying expensive ones prescribed by doctors. Even if they do buy more expensive medicines, they apply a degree of flexibility. According to a man:

‘Often I don’t use the whole strip as I recover back before completing the course… Here [local drug store] I can buy medicines according to my requirement… This helps me avoid unnecessary wastage of money and medicines.’ [user of drug stores because they are considered as economical]

#### Quality of treatment

The majority of the male respondents also judge the value of care based on the activities constituting treatment. It is observed that the real worth is evaluated only in the case of chronic illnesses (and not for common health problems). This stage involves the employment of different equipment and strategies for diagnosis and treatment, which men use as a parameter for determining the excellence of care. Many men perceive illness as something that lasts and has its impact on the body for a longer period, that it cannot be managed single-handily and essentially requires professional attention. This implies that men distinguish common health problems from illnesses. In this context, a man said: ‘It’s not an illness, but an indication that the body needs rest when you do heavy work.’ Hence, the question of judging the worth of care does not apply in the case of common illnesses that can be cured normally without professional assistance.

As most of the men in this study consider only one form of informal care (over-the-counter drugs) as being effective, they try to assess the quality of professionals as good or bad by comparing with other forms of informal care. The general perception of men is that professionals do not conduct their treatment on the basis of a superficial analysis (as informal healers do), rather based on a variety of technologies. Furthermore, professionals can show patients the specific affected area internally and its healing status post-treatment, which informal healers cannot. This assures men that they are undergoing correct treatment, thus in a way making professionals superior to informal healers. As remarked by a man:

‘At least we know for sure through TV [scanning monitor] that something is wrong with the body, but there [informal healing], we do not even know whether we are actually ill.’ [user of medical care]

#### Expected outcome of therapies

Men who are in work consider that time is best spent earning money and fulfilling families’ requirements, since they are the main source of income. As said by a man:

‘Meagre or no income when I am sick disrupts other aspects of life such as food, clothing, shelter, education etc. in the family.’ [user of medical care as perceived to have efficacy]

Therapies that cannot guarantee a quick recovery render men more vulnerable. This, in turn, puts them under stress, making it difficult to manage their financial position during sickness. As a man said:

‘I worry if I stay ill for long how my family will pay the costs of the situation.’ [user of clinical care]

Since different informal therapies (other than counter over-the-counter drugs) are considered time-consuming, with no indication with regard to the duration required for recovery, it appears natural that men seem less inclined towards such informal therapies, as observed by us during the study period. They gather such experiences by observing the situation of their wives or other female members of the family who usually approach informal healers, while their own experience with formal health care is that doctors usually indicate a tentative duration of recovery. As a man commented:

‘You will hear them [healers] often saying “I have done my duty… Now you have to wait patiently till it is cured and have faith in God”. Such things you won’t hear from doctors… My doctor usually says: “Within ten days you will get well, if not, come again”.’ [denounces informal healing as believed to have poor efficacy compared to clinical care]

Another reason why most men prefer formal care is that they believe it provides effective healing without causing further deterioration after an initial improvement. Compared to formal care, informal therapies for chronic illnesses can lead to frequent relapses, making men unfit for work aside from further hampering their livelihood. As one man shared:

‘Frequent illness will not let us start [working] and subsequently we will be kicked out from the workplace. So, we prefer to seek medical care that ensures full recovery besides preventing us from becoming sick within a short span… We can get back to work feeling more energetic… and can avoid being absent.’ [user of clinical care, considered as having good efficacy]

For some men, investing both time and money in a treatment (having already incurred economic losses) with no guarantee of a quick recovery can demotivate them totally to avail of that therapy. Men, therefore, need an assurance that the therapy is worth the effort. As a man said:

‘They [healers] test our patience and this is what we lack.’ [strong admirer and user of clinical care]

## Discussion

This study examined the health-seeking behaviour of men and women residing in an urban slum in Kolkata, India, by exploring the underlying perceptions affecting differences in their therapeutic choices. Previous findings had highlighted that gender differences manifested in men being more inclined towards formal health care, whereas women were more inclined towards alternative health care [15, 22]. Instead, the present analysis shows that both men and women make simultaneous use of formal and informal care. Yet, the key findings of this study show that they do so in different ways and have different motivations for their choices.

Women’s behaviour of mixing both formal and informal care indicates that they want to take care of their illness, but at the same time, they are keen on retaining their socio-cultural ethos, as reported in another study [49]. This health-seeking behaviour of women can be related to their lower (perceived) position in the community than men, as a result of which they have to face socio-cultural hurdles in terms of mobility, code of conduct, maintaining family prestige (by behaving well) and maintaining secrecy about their health problems.

Our study reveals that women benefit more from social support, as acknowledged in other studies [50–52]. Such social support is initiated through social integration with informal practitioners, who are distant kin or residing within the same neighbourhood. As shown in prior literature [53, 54] our study also confirms that such social association is developed through gender roles of maintaining social ties with close-knit kin (natal and consanguineous kin) [55] and another type of social relationships (neighbours and friends) [56].

Choices stem from financial stresses that women face both at familial (depending on their husbands for money) and institutional levels (high consultation charges and expensive medications) while seeking medical care. Our study reveals that men often avoid acknowledging health problems of women even when they are serious, as is found in other studies [57, 58]. Men who are guided by gender stereotypes in their views, according to which health is not considered as a core issue in life, think women are too sensitive to pain [59]. By consequence, they tend to dismiss and underestimate women’s need for professional intervention. Men’s judgement and control over women’s health is directly linked to their financial autonomy. Failure to persuade husbands often makes women turn towards informal health care. Women are forced to stick to informal care, even knowing it can be less effective. If absolutely necessary, they try to seek formal care that is inexpensive. This finding supports other studies that have shown poor and disadvantageous women are less likely to utilise formal health care services compared to affluent women [11] because of convenience, affordability and socio-cultural compatibility [60, 61].

The inability to participate meaningfully in formal health care use is also due to information asymmetry as regards how to adequately utilise health benefits. Women prefer door-to-door information on health benefits, as pointed out by another study [62]. In India, a major share of expenditure on health care is being borne out-of-pocket by the poor. This has resulted in after-treatment disasters such as selling or mortgaging assets and spending from savings, thus further worsening the poor’s financial conditions. Other findings point out that ignorance among the poor about free treatment and the complex and cumbersome procedures to obtain exemptions constrain the access of the poor to formal health care services [63].

Unlike women, men in the study area do not have to conform to societal gender stereotypes. Therefore, they have more freedom and flexibility in making their choices among therapies. However, their gender role is associated with higher adherence to formal health care. This might be explained by the fact that men identify themselves and are recognised as the earning members of the family. As a result, they cannot risk staying at home for long periods of time due to illness. These reasons drive men to choose formal care that primarily allows them to work (and helps in reducing the risk of circumstantial problems) by curing them fast and completely. As a consequence, the effectiveness of the cure constitutes the main lens through which men judge the value of the care received. Men’s choice for self-care is motivated only by cost-effectiveness. Illnesses are initially taken care of by using drugs from drug stores. Men’s judgement regarding choices of therapy is only based on time and treatment efficacy, needs that are both met by formal health care. During clinical encounters, men are more likely to communicate less and prefer quick diagnoses. This response might point towards masculine ideology, according to which men are neither meant to show nor expect emotions and sympathy [64], unlike women. Illness-related questions asked by clinical professionals make men comfortable; hence making professional care more preferable. This way, they feel can escape discussions about other social aspects, which are a potential consequence of the sympathetic exchange of words.

Another aspect of men’s unease related to informal healing is its indigenous concept of treatment, which is not scientific, is not aligned with medical or scholarly discourse, and include the application of rudimentary technology in health and healing, thus putting a question mark against the efficiency and reliability of treatment. However, further research is needed to ascertain how physicians in these two healing domains validate their therapies and to examine the actual accuracy of diagnosis in these two therapy domains.

Finally, when it comes to male/female choices of physicians and therapies, we found that women are sometimes moving back and forth between informal and formal health care or utilising both. Instead, men are generally static as regards their treatment choices. The reason might be that women bear more cultural demands, social responsibilities and economic consequences, whereas men bear only economic stressors. In order to adjust to these multiple factors, women state an ample number of reasons for making therapeutic choices compared to men. This may have implications for examining the appropriateness and suitability of treatment provided to women as compared to men. Further research is needed to assess the reasons for this apparent inconsistency or possibly perceived complementarity in using formal and informal therapies.

To conclude, it can be said that the notion of shortage may be a broader concept, which men and women residing in the urban slum of Kolkata experience and negotiate in a different way when it comes to the health care usage. Their very existence in society as immigrants exposes them to multi-faceted shortages in every aspect of life, the negative effect of which is also seen in their health-seeking behaviour. This suggests that medical adherence is secondary to the prevailing health care problems. Rather, policy approaches should be directed more towards effective communication, material access and awareness of social class in order to enhance users’ adherence.

## Conclusion

We find that both men’s and women’s choices of treatment are related to their daily interactions with society, which create either a favourable or unfavourable condition for seeking a treatment. Both men and women utilise informal and formal therapies, but often move back and forth between the two domains to adjust to their economic situation and socio-cultural norms. However, men believe in a complete cure so as to avoid endangering their gender role; women place more of an emphasis on avoiding social and economic penalties and therefore improvise by adhering to poor-quality therapies. These findings can by no means be generalised to other contexts, and even generalisation to other slums should be made with caution, as every slum settlement presents a unique religious, ethnic, linguistic, socio-cultural and socio-economic composition. Furthermore, the focus of this paper is on gendered health experiences. It is therefore possible that other traits influencing health care-seeking behaviour and interacting with gender have not been addressed in the present study.

## Footnotes

1 Only 24 households in the study area belong to the Christian religious group, out of which 18 households including men and women could be interviewed. Of the remaining six households, four were not ready for the interview and two were found closed during the study period. As a result, follow-up interviews with Christian female participants could not be carried out due to the scarcity of participants.

2
http://www.cehat.org/go/uploads/EthicalGuidelines/ethicalguidelines.pdf

3
http://www.anusandhantrust.org/centres/Ethics_in_health_research_-_A_social_science_perspective.pdf

4 Incest refers to having sexual relations with a close relative in the family, such as a brother, sister, uncle, aunt etc., whereas affiliate refers to the relationship formed by the treaty of marriage, such as a brother-in-law, sister-in-law, mother-in-law, father-in-law etc. Establishing sexual relationships with any of these members other than the spouse is considered adulterous.
